# PB.43. A clinical audit of breast cancer staging

**DOI:** 10.1186/bcr3727

**Published:** 2014-11-03

**Authors:** A Sulistijo, A Falamarzi, C Chen, C Lightner Ferrer, D Tan, THJ Kong, S O'Keeffe, J Kavanagh

**Affiliations:** 1 School of Medicine, Trinity College Dublin, Ireland; 2 St James's Hospital, Dublin, Ireland

## Introduction

Accurate local and distant staging of breast cancer is essential in determining appropriate disease management and prognosis. Staging investigations delay surgical management and increase cost and manpower requirements. The purpose of the study was to retrospectively assess the utility of imaging in breast cancer staging at St James's Hospital, Dublin between 2012 and 2013.

## Methods

The EPR was queried and 559 patients diagnosed with breast cancer between 2012 and 2013 were selected for inclusion in this study. The demographic characteristics, radiological (mammogram, US, MRI, CT), and histopathological records were reviewed. Microsoft Excel 2011 was used to analyse the data using descriptive statistics.

## Results

Of 559 patients (age range 19 to 93 years), 298 patients (53%) underwent staging CT TAP, triggering at least one further investigation in 134 patients (45%). Forty-three of these staged patients (14%) had distant metastases; 129 patients (43%) had normal axillary US, of whom five patients (4%) had distant metastases. In contrast, 22% of patients with abnormal axillary US had metastases. Five of 32 patients (16%) with triple-negative disease had metastases. Breast MRI was performed in 30% of patients, triggering further investigations in 37%. A further 250 investigations were initiated on the basis of CT/MRI in 559 patients.

## Conclusion

The excess use of staging CT in patients with early breast cancer and low axillary lymph node burden was not in compliance with the local protocol. Patients with triple-negative disease did not have a higher incidence of metastases at presentation. An average of one further investigation for every two patients is triggered by staging CT and MRI.

